# Haemostatic Action of Peptone in Typhoid Haemorrhages

**Published:** 1917-03

**Authors:** 


**Haemostatic Action of Peptone in Typhoid Haemorrhages.** P. Nolf, Soc. Biol., July 22, 1916, uses 10 c.c. of a 5% sterilized solution, every day or every other day, according to circumstances, and claims uniformly good results.

